# Coccidioidal Endophthalmitis in Immunocompetent Person, California, USA

**DOI:** 10.3201/eid1806.111765

**Published:** 2012-06

**Authors:** Michael L Cheng, Matthew Leibowitz, Edward Ha

**Affiliations:** University of California, Los Angeles, California, USA

**Keywords:** Coccidioides spp., coccidioides, endophthalmitis, voriconazole, coccidioidomycosis, eye enucleation, fungi, immunocompetent, California

**To the Editor:** In the United States, dimorphic fungi of the species *Coccidioides* are endemic to California (particularly the Central Valley), southern Arizona, southern New Mexico, and western Texas. Although there are a relatively large number of coccidioidomycosis cases in the United States (≈150,000/year), intraocular coccidioidomycosis is uncommon (*1**,**2*). We report a case of coccidioidal endophthalmitis in an immunocompetent person.

In October 2010, a 55-year-old white man in Santa Clarita, California, had severe pneumonia, drenching sweats, and an associated 25-pound weight loss. Three weeks later, when his symptoms had nearly resolved, the man reported having scratched his left eye with his eyeglasses and subsequent development of increasing redness, pain, and progressive vision loss (from 20/10 to 20/60 without correction).

In November 2010, the man sought the care of an ophthalmologist, who noted panuveitis of the left eye. Laboratory testing was performed: the erythrocyte sedimentation rate was 49 mm/h (reference 0–22 mm/h), and test results were negative for human leukocyte antigen B27, angiotensin-converting enzyme, rapid plasma reagin, and antinuclear antibody. The patient was started on topical corticosteroids and escalated to high-dose prednisone soon thereafter without improvement. Pain continued to increase in his left eye, and visual acuity declined to hand motion only.

Thus, in February 2011, the patient was referred to our institution, where an ocular ultrasound showed vitreous opacities (Figure). He underwent vitrectomy with intravitreal injection of empiric antimicrobial drugs, including voriconazole. Aqueous fluid obtained intraoperatively grew mold, and the patient was admitted to the hospital for systemic antifungal therapy.

**Figure:**
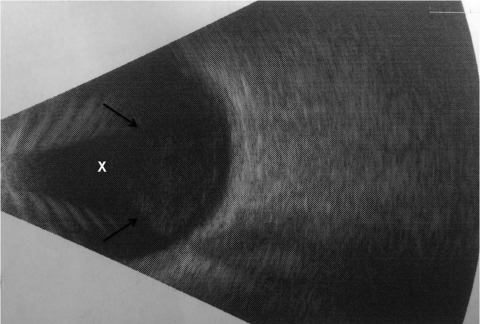
Ocular ultrasound demonstrating hyperechoic, punctate opacities (arrows) within the vitreous chamber (X) of a patient with coccidioidomycosis, California, USA.

The patient’s history was unremarkable except for avid mountain biking in the Central Valley of California. His physical examination was notable for left visual acuity limited to hand motion only, limited extraocular movement, conjunctival injection, and hypopyon. His HIV test result was negative. Computed tomography (CT) scanning of the chest showed micronodules in the right upper lobe, suggesting previous pulmonary coccidioidal infection. Intravenous voriconazole (4 mg/kg every 12 hours) was administered along with daily intravitreal injections of voriconazole while the patient was hospitalized. Results for coccidioidal antibody testing were positive by enzyme immunoassay and immunodiffusion but negative by serum complement fixation. Nucleic acid hybridization testing of aqueous fluid cultures identified *Coccidioides* spp. Results of a CT brain scan, lumbar puncture, and bone scan were normal.

After 1 week of hospitalization, the patient was discharged on oral voriconazole (4 mg/kg 2×/day). Because of transaminitis, the patient was transitioned to fluconazole (800 mg/day) 4 weeks later. He underwent 13 subsequent intravitreal injections of amphotericin and voriconazole. Eleven months after discharge, the patient’s best-corrected visual acuity was 20/25, and his ocular media were clear and without any lesions.

Coccidioidomycosis often goes undetected because up to 60% of affected patients are asymptomatic (*3*). When signs and symptoms are present, they vary from subclinical infection to acute pneumonia to disseminated disease (*3*). The rate of extrapulmonary complications is estimated at 0.5% of infections in white persons, but such complications may occur in 30%–50% of immunosuppressed patients with coccidioidomycosis (*3*). Disseminated coccidioidomycosis typically involves the skin, meninges, or bone (*3*); however, intraocular involvement has also been described (*1*). A review of the literature shows 25 reported cases of intraocular coccidioidomycosis. When present, intraocular involvement is associated with serious consequences, frequently leading to eye enucleation; 1 case series described eventual enucleation in 50% of reported patients who did not die from disseminated coccidioidal infection (*2*).

For the patient in our report, in the setting of reported trauma and negative metastatic work-up results, it is unclear whether ocular disease resulted independently as an exogenous infection or from endogenous lymphatic and/or hematogenous spread from the patient’s lung. Diagnosis of coccidioidal endophthalmitis can be difficult, often relying on serum or nonocular tissue evaluation (*4*). Intraocular coccidioidal involvement usually occurs with widespread infection (*5*). Thus, even with apparent isolated ocular findings, evaluation for disseminated disease is warranted, including a careful history and physical examination, CT chest scan, bone scan, intracranial imaging, and lumbar puncture. Evaluation for immunosuppression, including HIV status, is warranted.

The optimal systemic antifungal therapy for intraocular coccidioidal infection is unclear, although fluconazole is the drug of choice for extrapulmonary coccidioidomycosis, including meningitis (*3*). Fluconazole has good ocular penetration; however, voriconazole also achieves excellent intraocular levels (*6*) at lower 90% minimum inhibitory concentration levels (*7*). Furthermore, Gabrielian and Hariprasad (*8*) described an immunocompetent patient with treated and stable nonocular disseminated coccidioidomycosis who showed development of new vitritis and choroiditis 8 weeks into high-dose fluconazole therapy; his intraocular disease resolved within 2–4 weeks of transition to voriconazole.

The patient in our report received systemic voriconazole for 4 weeks plus repeated intravitreal voriconazole injections on follow-up. It is possible that this initial therapy had an effect on his positive outcome and the avoidance of eye enucleation. The optimal length of therapy is unclear; however, this patient will receive prolonged treatment (>1 year) with high-dose fluconazole, followed by a slow taper guided by serologic testing and regular ophthalmologic examination. Future research should evaluate which antifungal therapy is superior and the appropriate duration of treatment.
